# Response to the Letter by Guagliardo et al

**DOI:** 10.1093/ofid/ofag427

**Published:** 2026-07-11

**Authors:** Caroline B Ewing, Dawd Siraj, Alexander Lepak

**Affiliations:** Division of Infectious Disease, Department of Medicine, University of Wisconsin, Madison, Wisconsin, USA; Division of Infectious Disease, Department of Medicine, University of Wisconsin, Madison, Wisconsin, USA; Division of Infectious Disease, Department of Medicine, University of Wisconsin, Madison, Wisconsin, USA

To the editor—We appreciate the opportunity to respond to the letter by Guagliardo et al. regarding our recently published case report [1, 2]. Our intent is to clarify several points raised and restate the clinical context in which the report was developed.

First, by definition, a case report is a description of the clinical evaluation, diagnosis, and management of an individual patient, written by clinicians for the benefit of other clinicians. It is not intended to constitute a public health investigation, nor did we characterize it as such. Expecting a case report to comprehensively review all aspects of a disease represents a misunderstanding of its purpose and scope.

The clinical description of the patient's history was presented accurately by the clinicians directly involved in her care. We respectfully disagree with the assertion that our interpretation of the patient's clinical presentation was inaccurate. While we recognize and value the epidemiologic expertise of Dr. Guagliardo and colleagues, our training and clinical experience in medicine and infectious diseases provide the necessary foundation for interpreting clinical findings. Importantly, we personally evaluated the patient, obtained the history in real time, performed the physical examinations, and followed her longitudinally—information that was not available to the authors of the letter. We spent many hours face-to-face with the patient and family using open-ended, unscripted interviewing techniques getting detailed accounts of her activities and symptoms. A scripted history obtained by telephone by an individual not involved in the patient's clinical care occurring weeks after the patient's acute presentation cannot be considered equivalent to bedside evaluation during the illness. It is not surprising that details may differ between these methods due to recall bias, but we stand confidently behind our detailed account of the clinical case.

The definition of biphasic illness referenced by Guagliardo et al. is also inaccurate. Biphasic illness does not refer to relapse or recurrence of similar symptoms. As defined in infectious diseases literature, biphasic illness describes two distinct phases of symptoms separated by a period of improvement or relative remission [3–9]. The first phase often includes nonspecific symptoms such as fever, headache, myalgia, and fatigue; the second phase may involve recurrence or progression of symptoms, often with distinct or more severe manifestations. Examples of biphasic illnesses in infectious disease include several arboviral and bacterial illnesses (e.g., dengue, *Borrelia* infections, Japanese encephalitis, and leptospirosis). Oropouche virus has similarly been referenced in this manner [7, 10]. The acute phase typically presents with fever, headache, myalgia, arthralgia, and other influenza-like symptoms. After the initial symptoms resolve, patients experience a symptom-free interval before entering a second phase. Our case description was completely consistent with the description of a biphasic illness and with previous Oropouche virus cases.

With respect to public health guidance, we note that the recommendations cited by Guagliardo et al. were already included in our report. Additionally, we note the table included in their letter does not align with the CDC website referenced.

The diagnostic pathway described in our report was accurate. The clinician involved in the patient's care appropriately recommended Chikungunya IgM testing, which ultimately led the Wisconsin State Laboratory of Hygiene and the Wisconsin Department of Health Services to refer the specimen to the CDC Arboviral Diseases Laboratory for confirmatory testing for chikungunya and Oropouche viruses. These specialized tests are not directly available in our health system. Throughout the evaluation, we discussed all available testing options with our public health partners, including those referenced by Guagliardo et al.

Finally, we reiterate that discussions on mechanisms of disease, transmission, or pathogenesis fall outside the scope of a case report. Readers seeking comprehensive reviews of Oropouche virus may consult the literature cited in our paper, including publications authored by Guagliardo et al. We remain grateful for the critical role of state and federal public health laboratories in facilitating specialized diagnostic testing. Nonetheless, the diagnosis was made by the treating clinicians who directly evaluated, examined, and followed the patient across inpatient and outpatient settings.

In closing, we believe it is important to address the broader context of the concerns raised by Guagliardo et al. Although we value the essential contributions of state and federal public health laboratories on a daily basis, the criticisms expressed in their letter are disproportionate to the scope and intent of our report, and an unwarranted challenge to the clinicians who provided direct patient care. We stand by the accuracy and integrity of our clinical description, our interpretation of the patient's presentation, and the diagnostic assessment as documented in our case report.
